# Chemoradiation treatment with or without concurrent tumor-treating fields (TTFields) therapy in newly diagnosed glioblastoma (GBM) patients in China

**DOI:** 10.1186/s41016-025-00391-w

**Published:** 2025-03-07

**Authors:** Liping Liang, Lingchao Chen, Chunxia Ni, Wenyin Shi, Zhirui Zhou, Shu Chen, Wenjia Zhu, Jiabing Liu, Xianxin Qiu, Wanzun Lin, Junyan Zhang, Zhiyong Qin, Yang Wang

**Affiliations:** 1 Department of Radiation Oncology Center, Huashan Hospital, Fudan University, Shanghai, 200040 China; 2 Department of Neurosurgery, Huashan Hospital, Fudan University, Shanghai, 200040 China; 3 Department of Radiation Oncology, Shanghai Gamma Hospital, Shanghai, 200235 China; 4 Department of Radiation Oncology, Sidney Kimmel Cancer Center, Thomas Jefferson University, Philadelphia, PA USA; 5 Department of Radiation Oncology, Shanghai Proton and Heavy Ion Center, Fudan University Cancer Hospital, Shanghai, 201321 China; 6 Department of Clinical Epidemiology and Evidence-Based Medicine, Shanxi Bethune Hospital, Shanxi Academy of Medical Sciences, Taiyuan, China

**Keywords:** TTFields therapy, Glioblastoma, Concurrent therapy, Chemoradiotherapy

## Abstract

**Background:**

Tumor-treating fields (TTFields) therapy and radiotherapy may have synergistic anti-glioma effect based on preclinical studies. The combination of chemoradiation therapy (CRT) with TTFields therapy has noticeably attracted clinicians’ attention. This study aimed to provide insights into the clinical outcomes of patients with newly diagnosed glioblastoma who received either concurrent CRT and TTFields therapy or adjuvant TTFields therapy following CRT. The findings were based on a cohort of patients who were treated at Huashan Hospital (Shanghai, China).

**Methods:**

This retrospective study analyzed ndGBM patients’ clinical outcomes who were treated at Huashan Hospital and received TTFields therapy. Patients were categorized into two groups: one group received adjuvant TTFields therapy after completing CRT (referred to as the A-TTF group), while the other received TTFields therapy concurrently with CRT and continued TTFields after treatment (referred to as the CA-TTF group). The study evaluated treatment efficacy and toxicities, comparing outcomes between the two groups. Overall survival (OS) and progression-free survival (PFS) were analyzed using the Kaplan–Meier method. To mitigate confounding factors, efficacy was assessed using the Cox proportional hazards regression model, propensity score matching, and inverse probability of treatment weighting (IPTW) based on the propensity score.

**Results:**

A total of 72 patients with ndGBM were included in the study. Among them, 41 patients received concurrent and adjuvant TTFields therapy in combination with CRT (CA-TTF group), and 31 patients received adjuvant TTFields therapy with temozolomide (A-TTF group). The median follow-up time was 18.0 months. No significant differences were observed in median PFS (14.2 vs. 15.0 months, *P* = 0.92) or OS (20.8 vs. 20.0 months, *P* = 0.92) between the CA-TTF and A-TTF groups. Skin toxicity was common, while manageable, with no significant difference between the two groups. Following IPTW adjustment, the hazard ratios for PFS and OS indicated a potential advantage for the CA-TTF group, although this difference was not statistically significant.

**Conclusion:**

Concurrent CRT and TTFields therapy emerged safe for newly diagnosed GBM patients. Although no significant survival differences were found between the CA-TTF and A-TTF groups, the potential benefit of concurrent TTFields warrants further investigation through large-scale clinical trials.

**Supplementary Information:**

The online version contains supplementary material available at 10.1186/s41016-025-00391-w.

## Background

Glioblastoma (GBM), characterized by its extensive invasiveness and dismal prognosis, is the most prevalent primary malignant brain tumor in adults [1]. Tumor-treating fields (TTFields) therapy is a novel anti-mitotic therapeutic modality that utilizes low-intensity, intermediate-frequency electric fields to inhibit cell proliferation and disrupt cancer cell replication [2]. It was approved for patients with newly diagnosed and recurrent GBM [3–7]. The EF-14 clinical trial demonstrated that adding TTFields to maintenance temozolomide chemotherapy following radiotherapy (RT) significantly improved the progression-free survival (PFS) and overall survival (OS) rates in patients with newly diagnosed GBM (ndGBM). Consequently, TTFields plus temozolomide was recommended as the first-line standard adjuvant therapy for patients with ndGBM following completing RT [5, 6].

Several studies have investigated the mechanisms of TTFields therapy. Besides its anti-mitotic effect, TTFields therapy has also been found to have other mechanisms of action, such as increasing cell permeability, activating autophagy, stimulating immune response, and inhibiting DNA damage repair [8]. A preclinical study indicated that the combination of TTFields and irradiation has a synergistic anti-glioma effect by inhibiting the repair of radiation-induced DNA damage. When glioma cells were treated with TTFields therapy after RT, more than 40% of the initial DNA damage remained unrepaired, as assessed by the comet assay. After TTFields treatment, the BRCA1 DNA-damage response was significantly downregulated and DNA double-strand break (DSB) repair was reduced. In addition to its synergy with radiation, TTFields therapy has been shown to enhance the effects of chemotherapy by increasing drug delivery to cancer cells, disrupting cell division, and sensitizing glioblastoma cells to temozolomide [9]. These results provided a strong rationale for the application of the combination of concurrent TTFields with chemoradiation therapy (CRT) [10–14] . Consequently, early clinical trials were conducted to evaluate the safety and feasibility of this combination therapy. The SPARE trial was the first study to report the feasibility of concurrent TTFields with CRT in patients with ndGBM. While skin toxicities were common, they were mild and well-tolerated, and 83.3% of patients experienced grade 1 or 2 skin-related adverse events (AEs). The median PFS and OS were 9.3 months and 15.8 months, respectively, which compare favorably to historical benchmarks [15, 16]. Bokstain et al. also conducted a phase 1/phase 2 study, reporting the safety and feasibility concurrent TTFields therapy and RT in 10 patients during the phase 1. The findings were similar, and the majority of patients (80%) experienced mild-to-moderate skin toxicities (grade 1–2) caused by TTFields therapy. There was no grade 3 or higher toxicities, and the combination was considered well-tolerated. The median PFS was 8.9 months, while the median OS was not reached [17]. According to these two early phase trials, an international phase 3 randomized trial (EF-32, NCT04471844) is currently enrolling participants to compare concurrent TTFields versus maintenance TTFields only [18]. However, the results have not yet been reported.

This retrospective study aimed to determine the clinical benefits of concurrent and adjuvant TTFields therapy with chemoradiotherapy (CA-TTF group) versus adjuvant TTFields therapy after chemoradiotherapy only (A-TTF group) in our real clinical practice. A cohort of patients with ndGBM treated at Huashan Hospital (China) between 2020 and 2021 was reviewed. The primary experiences and findings regarding these two regimens were presented.

## Methods

### Patients

This retrospective study included ndGBM patients who underwent RT and TTFields treatment in Huashan Hospital Affiliated to Fudan University (Shanghai, China) between January 2020 and December 2021. Patients were categorized into two groups: one group received adjuvant TTFields therapy after completing CRT (referred to as the A-TTF group), while the other received TTFields therapy concurrently with CRT and continued TTFields after treatment (referred to as the CA-TTF group). The grouping decisions were made by clinicians based on the patient’s performance status (e.g., KPS score), tumor characteristics, and the patient’s willingness to undergo concurrent TTFields therapy during chemoradiation. All the included patients met the following criteria: (1) patients who aged 18 years or older with newly diagnosed *IDH1/2* wild-type GBM; (2) patients who underwent TTFields treatment for more than four weeks; (3) patients who completely underwent the standard Stupp regimen CRT. Patients were administered temozolomide concurrently with RT at a dose of 75 mg/m^2^ daily for 6 weeks. Following the concurrent phase, during the adjuvant phase, temozolomide was given at a dose of 150–200 mg/m^2^ for the first 5 days of each 28-day cycle, accounting for a total of 6 cycles. This study was approved by the Ethics Committee of Huashan Hospital Affiliated to Fudan University (Approval No. KY2023-1007).

Patients’ baseline characteristics, such as age, sex, Karnofsky performance status (KPS) score, the extent of resection, *MGMT* promoter methylation status, *TERT* promoter methylation status, TTFields usage, AEs, grade evaluation, PFS, and OS were collected. The extent of resection was classified as gross tumor resection (GTR), subtotal resection (STR), and biopsy [19].

### RT

All patients were immobilized in a supine position using a thermoplastic mask. In the CA-TTF group, a customized 5-mm thick latex-free open-cell styrene butadiene rubber foam was placed under the mask to accommodate the TTFields transducer arrays. Treatment planning computed tomography (CT) scan was performed for all patients without TTFields arrays and fused with post-operative magnetic resonance imaging (MRI). The target volumes were contoured according to the Radiation Therapy Oncology Group (RTOG) guidelines. The radiation prescription was 60 Gy in 30 fractions for all patients. In the CA-TTF group, the scalp, which was defined in the SPARE trial as the 5-mm thick area extending from the skin surface above the level of the foramen magnum, was accurately contoured and included as an organ at risk (OAR) in radiation planning. The scalp constraints were also adopted from the SPARE trial [15], which were summarized as follows: mean < 20 Gy, D20cc < 50 Gy, D30cc < 40 Gy. RT plans for all patients were generated using eclipse v15.5 (Varian, Palo Alto, CA, USA). Treatment was administered using Truebeam (Varian, Palo Alto, CA), with weekly verification via CBCT.

In the initial phase of the present study, TTFields arrays were removed daily for the first five patients to closely monitor skin conditions and ensure safety. However, after observing no significant adverse effects on skin and reviewing findings from the SPARE study, the protocol was adjusted. For subsequent patients, arrays were not removed daily during the RT, promoting a more continuous application of TTFields therapy.

### TTFields therapy

In the CA-TTF group, TTFields therapy commenced within one week of starting RT for all patients. In the A-TTF group, treatment began 4–7 weeks following completing RT. Follow-up assessments began immediately postoperatively for both groups. Array placement alternated between two sites with each change. Monthly device logs and average daily use (ADU) were recorded for all patients. The physician and/or patient/caregiver examined the scalp reaction during transducer array replacement [15].

### Toxicity

Scalp toxicity was graded using the Common Terminology Criteria for Adverse Events (CTCAE, ver. 5.0) [20].

### Statistical analysis

Continuous variables were analyzed using Student’s *t*-test or Wilcoxon rank-sum test. Baseline categorical variables were analyzed by chi-squared or Fisher’s exact test. The propensity dataset was utilized to generate the inverse probability of treatment weighting (IPTW) dataset, which balanced all observable characteristics of patients in the CA-TTF group for making comparison with the A-TTF group.

The Kaplan–Meier method and the multivariate Cox regression model were used for survival analysis. The IPTW dataset was analyzed with the Cox regression model for sensitivity analysis. The results were expressed as adjusted hazard ratios (HRs) with 95% confidence intervals (95% CIs). The R 4.2.0 software (http://cran.rproject.org, accessed on 1st May 2022) was utilized for statistical analysis.

## Results

### Study population

A total of 72 patients were included in this retrospective study. Among them, 41 patients received concurrent and adjuvant TTFields in combination with chemoradiotherapy (CA-TTF group), and 31 patients underwent adjuvant TTFields therapy after chemoradiotherapy (A-TTF group). All patients had GBM, were *IDH* wild type, and were classified as the WHO grade 4. Patients’ characteristics were well balanced between the two groups (Table 1). In the CA-TTF group, 31.7% of patients underwent STR or biopsy versus 25.8% in the A-TTF group. The *TERT* promoter mutation rate was 63.4% in the CA-TTF group versus 41.9% in the A-TTF group. The *MGMT* promoter methylation rate was 36.6% in the CA-TTF group versus 32.3% in the A-TTF group (Table 1).

**Table 1:** Patient characteristics

	TTFields		*P*-value
	CA-TTF (*N* = 41)	A-TTF (*N* = 31)	
**Sex**			
Female	13 (31.7%)	13 (41.9%)	0.518
Male	28 (68.3%)	18 (58.1%)	
**Age (years)**			
Median [Min, Max]	53.0 [22.0, 76.0]	48.0 [19.0, 74.0]	0.615
**Extent of resection**			
GTR	28 (68.3%)	23 (74.2%)	0.777
STR/Biopsy	13 (31.7%)	8 (25.8%)	
**MGMT promoter methylation**			
Methylated	15 (36.6%)	10 (32.3%)	0.907
Unmethylated	24 (58.5%)	19 (61.3%)	
Unknown	2 (4.9%)	2 (6.5%)	
**TERT promoter mutation status**			
Wild-type	12 (29.3%)	14 (45.2%)	0.192
Mutation	26 (63.4%)	13 (41.9%)	
Unknown	3 (7.3%)	4 (12.9%)	
**Baseline KPS**			
Median [Min, Max]	90.0 [60.0, 90.0]	80.0 [60.0, 90.0]	0.443
**Compliance with TTFields (hours/day)**			
Median [Min, Max]	21.1 [8.88, 23.0]	21.6 [15.8, 23.3]	0.491
**Duration of TTFields (months)**			
Median [Min, Max]	10.0 [1.00, 31.0]	10.4 [1.00, 35.0]	0.203

*GTR* gross tumor resection, *STR* subtotal resection

### Compliance and duration of TTFields therapy

In the CA-TTF group, the initial 5 patients had TTField arrays removed daily during radiation delivery, while the rest kept the arrays on during RT. Scalp-sparing RT was administered to 35 (83.3%) patients. Both groups achieved over 75% compliance with TTFields therapy (18 h per day) [21]. The median compliance was 21.1 h per day for the CA-TTF group, and 21.6 h per day for the A-TTF group. The median durations of TTFields therapy were similar, at 10.0 months for the CA-TTF group and 10.4 months for the A-TTF group (Table 1).

### Toxicity

Skin-related AEs were assessed in this study. These events included dermatitis, pruritus, electric sensation, and skin burning sensation. Dermatitis, which included scalp irritation, dry skin, folliculitis, erythema, color change, or rash, was similar in both groups, in which grade 1 AEs occurred in 21.95% of patients in the CA-TTF group and in 25.81% of patients in the A-TTF group. Grade 2 AEs were found in 34.15% of patients in the CA-TTF group and in 32.26% of patients in the A-TTF group. Grade 3 skin-related AEs were rare, occurring only in 2.44% of patients in the CA-TTF group and in 3.23% of patients in the A-TTF group. Pruritus, another skin-related AE, was noted in both groups. Grade 1 AEs were identified in 9.76% of patients in the CA-TTF group and in 9.68% of patients in the A-TTF group. Grade 2 AEs were less frequent, affecting 4.88% of patients in the CA-TTF group and 3.22% of patients in the A-TTF group. Electric sensation occurred in 2.44% of patients in the CA-TTF group. Skin burning sensation, another infrequent event, was reported in both groups, in which grade 1 AEs documented in 2.44% of patients in the CA-TTF group and in 3.22% of patients in the A-TTF group (Table 2).

**Table 2:** TTFields-related adverse events

	**CA-TTF (*N* = 41)**	**A-TTF (*N* = 31)**
**Skin AEs, *n* (%)**		
Dermatitis^a^		
Grade 1 AEs, *n* (%)	9 (21.95%)	8 (25.81%)
Grade 2 AEs, *n* (%)	14 (34.15%)	9 (32.26%)
Grade 3 AEs, *n* (%)	1 (2.44%)	1 (3.23%)
Pruritus		
Grade 1 AEs, *n* (%)	4 (9.76%)	3 (9.68%)
Grade 2 AEs, *n* (%)	2 (4.88%)	1 (3.22%)
Electric sensation		
Grade 1 AEs, *n* (%)	1 (2.44%)	
Skin burning sensation		
Grade 1 AEs, *n* (%)	1 (2.44%)	1 (3.22%)

^a^Dermatitis included scalp irritation, dry skin, folliculitis, erythema, color change, or rash

### Survival outcomes

**Fig. 1:**
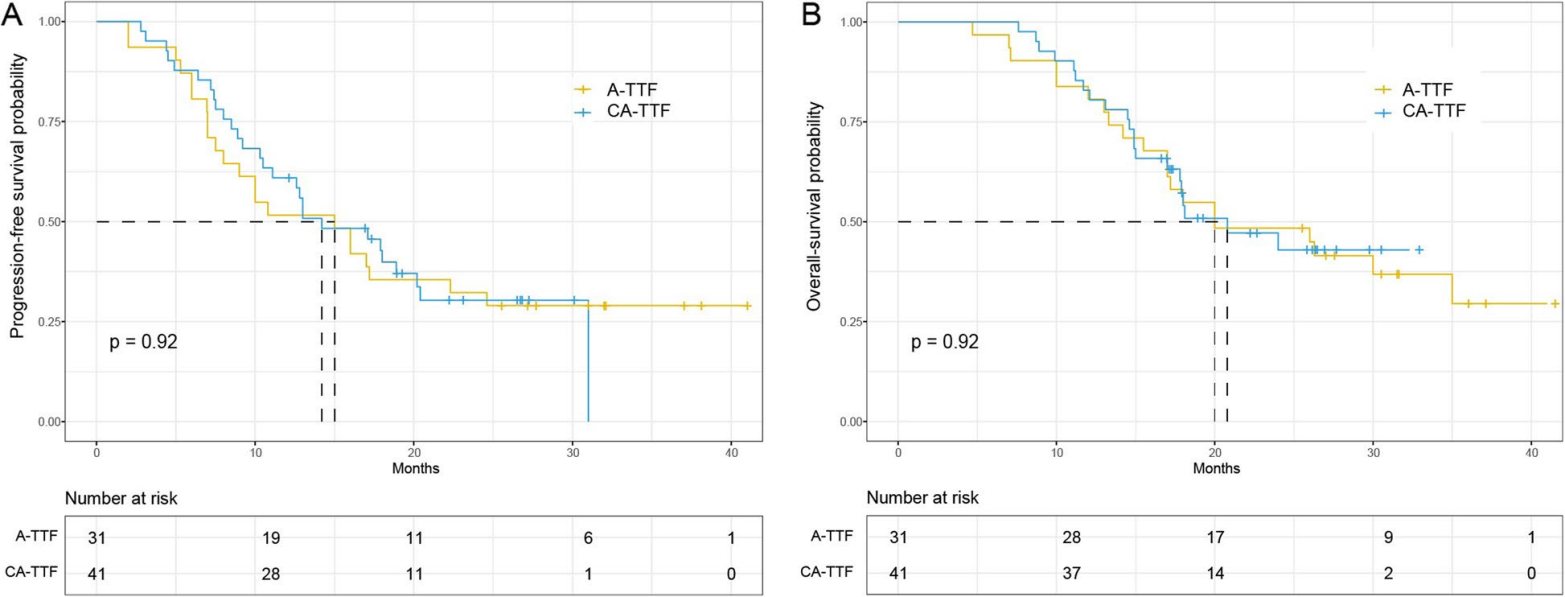
Comparison of progression-free survival (**A**) and overall survival (**B**) between CA-TTF and A-TTF groups

There was no significant difference in median progression-free survival (PFS) between the two groups. The PFS was 14.2 months (95% CI: 11.1–20.4) in the CA-TTF group and 15.0 months (95% CI: 8.0–NA) in the A-TTF group, with a proportional HR of 0.97 (95% CI: 0.55–1.70, *P* = 0.92) (Fig. 1). Similarly, there was no significant difference in OS between the two groups, with 20.8 months (95% CI: 17.8–NA) in the CA-TTF group versus 20.0 months (95% CI: 17.0–NA) in the A-TTF group, with an HR of 0.97 (95% CI: 0.51–1.80, *P* = 0.92) (Fig. 1). In the CA-TTF group, the 1-year PFS and OS rates were 61.0% (95% CI: 47.7–77.9%) and 82.9% (95% CI: 72.2–95.3%), respectively. Conversely, the A-TTF group demonstrated a 1-year PFS rate of 51.6% (95% CI: 36.7–72.6%) and a 1-year OS rate of 80.6% (95% CI: 67.9–95.8%) (Fig. 1). After conducting IPTW to balance the baseline characteristics between the CA-TTF and A-TTF groups, there remained no significant differences in PFS and OS. However, the adjusted HR for PFS decreased from 0.97 (95% CI: 0.55–1.70, *P* = 0.92) to 0.77 (95% CI: 0.44–1.30, *P* = 0.344), and the adjusted HR for OS decreased from 0.97 (95% CI: 0.51–1.80, *P* = 0.92) to 0.74 (95% CI: 0.40–1.37, *P* = 0.336) for OS (Table 3).

**Table 3:** PFS and OS analysis after inverse probability treatment weighting (IPTW)

PFS				
TTFields concurrent	HR	*P* value	95% CI	
			Lower	Upper
	0.77	0.344	0.44	1.3
OS				
TTFields concurrent	HR	*P* value	95% CI	
			Lower	Upper
	0.74	0.336	0.4	1.37

In Cox regression analysis for CA-TTF group, four patients were excluded from the analysis due to missing molecular status data. Univariate analysis revealed that the duration of TTFields had a significant impact on OS (HR = 0.93, 95% CI: 0.87–0.99, *P* = 0.019), this effect remained significant in multivariate analysis (HR = 0.89, 95% CI: 0.81–0.98, *P* = 0.013). In both univariate and multivariate Cox regression analyses, male patients exhibited shorter OS with HRs of 3.72 (95% CI: 1.08–12.79, *P* = 0.037) and 4.74 (95% CI: 1.12–20.09, *P* = 0.035), respectively. Similarly, for PFS, male patients also showed significantly reduced survival in both univariate and multivariate analyses, with HRs of 3.29 (95% CI: 1.22–8.88, *P* = 0.019) and 3.63 (95% CI: 1.18–11.18, *P* = 0.025), respectively. Subtotal resection was associated with shorter PFS and OS compared to gross total resection in multivariate analyses, with HRs of 5.25 (95% CI: 1.47–18.91, *P* = 0.011) and 3.37 (95% CI: 1.00–11.32, *P* = 0.050), respectively. Multivariate Cox regression analysis also revealed a significant association between longer patient compliance with TTFields (hours/day) and extended PFS with a hazard ratio (HR) of 0.68 (95% CI: 0.49–0.93, *P* = 0.016). Both univariate and multivariate analyses showed longer duration of TTFields accompanying with better OS, with HRs of 0.93 (95% CI: 0.87–0.99, *P* = 0.019) and 0.89 (95% CI: 0.81–0.98, *P* = 0.013), respectively, as indicated in Table 4.

**Table 4:** Univariate and multivariate analyses of progression-free survival (PFS) and overall survival (OS) in CA-TTF group patients

			Univariate (95% CI, crude *P* value)		Multivariate (95% CI, crude *P* value)	
		*n* (%)	PFS	OS	PFS	OS
**Age**	Median (Min, Max)	53.0 (22.0,76.0)	1.01 (0.98–1.04, *p* = 0.523)	1.04 (1.00–1.07, *p* = 0.048)	1.01 (0.96–1.07, *p* = 0.670)	1.01 (0.94–1.09, *p* = 0.691)
**Sex**	Female	11 (29.7)	-	-	-	-
	Male	26 (70.3)	3.29 (1.22–8.88, *p* = 0.019)	3.72 (1.08–12.79, *p* = 0.037)	3.63 (1.18–11.18, *p* = 0.025)	4.74 (1.12–20.09, *p* = 0.035)
**Extent of resection**	GTR	26 (70.3)	-	-	-	-
	STR/Biopsy	11 (29.7)	2.13 (0.92–4.89, *p* = 0.076)	1.65 (0.67–4.06, *p* = 0.274)	5.28 (1.47–18.91, *p* = 0.011)	3.37 (1.00–11.32, *p* = 0.050)
**MGMT promoter region methylation**	Methylated	15 (40.5)	-	-	-	-
	Unmethylated	22 (59.5)	1.77 (0.76–4.12, *p* = 0.183)	1.40 (0.56–3.51, *p* = 0.477)	2.34 (0.79–6.95, *p* = 0.125)	3.57 (0.88–14.53, *p* = 0.076)
**TERT promoter mutation status**	Mutation	25 (67.6)	-	-	-	-
	Wild-type	12 (32.4)	0.51 (0.20–1.28, *p* = 0.152)	0.85 (0.34–2.14, *p* = 0.730)	0.32 (0.08–1.26, *p* = 0.104)	0.39 (0.06–2.43, *p* = 0.311)
**Baseline KPS ≥ 90**	NO	11 (29.7)	-	-	-	-
	YES	26 (70.3)	0.64 (0.28–1.44, *p* = 0.280)	0.45 (0.19–1.09, *p* = 0.077)	1.29 (0.41–4.00, *p* = 0.665)	0.53 (0.14–1.98, *p* = 0.346)
**Patient compliance with TTF (hours/day)**	Median (Min, Max)	20.12 (16.8,23.0)	0.82 (0.61–1.10, *p* = 0.191)	0.93 (0.68–1.29, *p* = 0.669)	0.68 (0.49–0.93, *p* = 0.016)	1.03 (0.70–1.52, *p* = 0.872)
**Duration of TTF(months)**	Median (Min, Max)	10.0 (1.0,31.0)	0.97 (0.93–1.02, *p* = 0.260)	0.93 (0.87–0.99, *p* = 0.019)	0.94 (0.86–1.03, *p* = 0.203)	0.89 (0.81–0.98, *p* = 0.013)

**Fig. 2:**
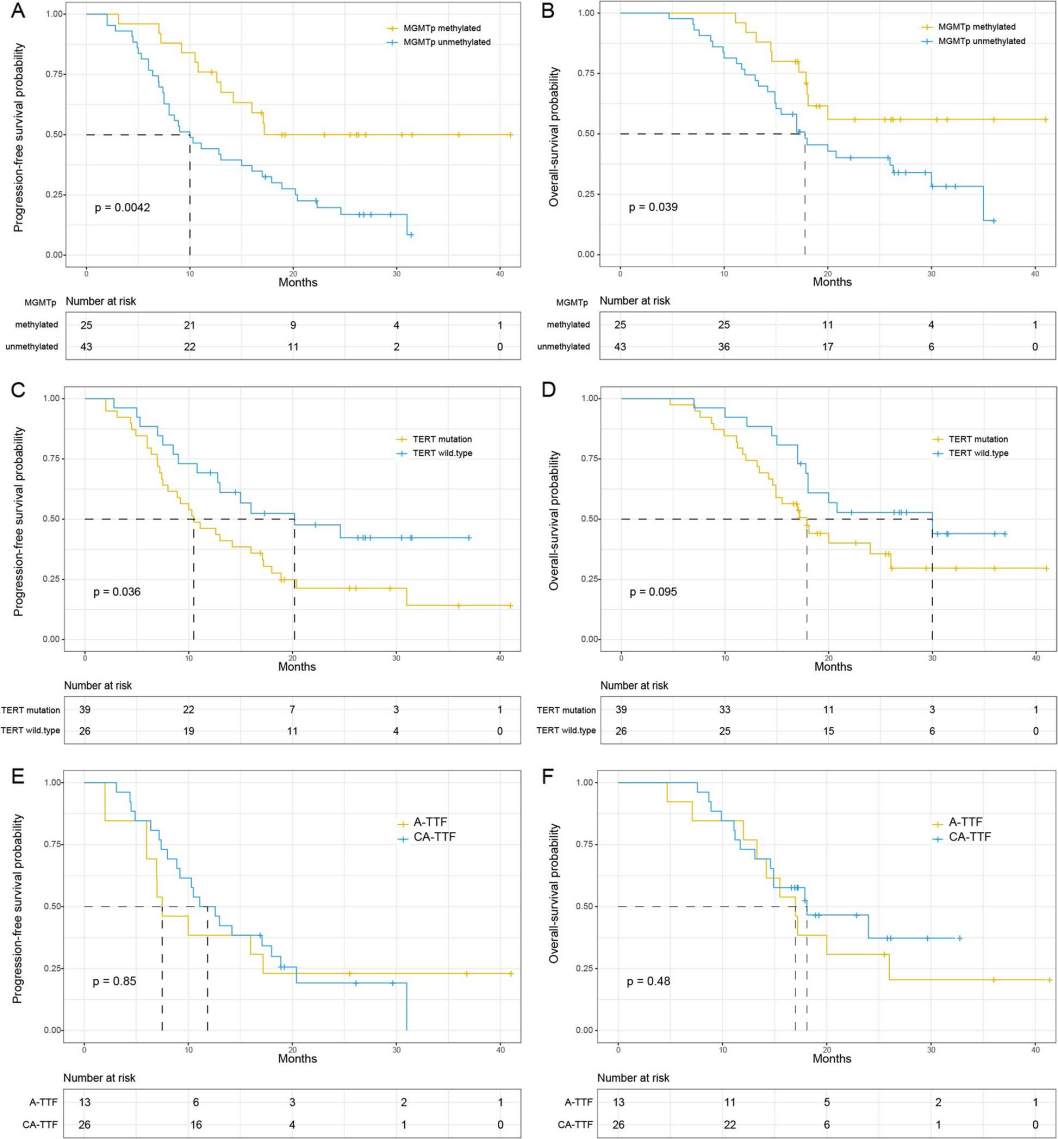
Influences of *MGMT* promoter methylation (**A**, **B**), *TERT* status (**C**, **D**), and *TERT* mutation (**E**, **F**) on progression-free survival and overall survival

In the entire cohort, MGMT promoter methylation status was found to be associated with PFS and OS. The median PFS for the *MGMT* promoter methylated group was indeterminate (95% CI: 14.2–NA), while it was 10 months for the *MGMT* promoter unmethylated group (95% CI: 7.5–17.9, *P* = 0.0042) (Fig. 2A). The median OS was indeterminate for the *MGMT* methylated group (95% CI: 18.0–NA), whereas it was 17.8 months for the unmethylated group (95% CI: 14.9–30, *P* = 0.039) (Fig. 2B).

The median PFS for the *TERT* mutation group was 10.5 months (95% CI: 8.0–17.2), while it was 20.0 months for the *TERT* wild-type group (95% CI: 12.8–NA, *P* = 0.036) (Fig. 2C). The median OS for the *TERT* mutation group was 17.9 months (95% CI: 14.9–NA), whereas it was 30.0 months for the wild-type group (95% CI: 18.0–NA, *P* = 0.095) (Fig. 2D). On the other hand, in patients with *TERT* mutations, there was no difference in PFS or OS between CA-TTF and A-TTF groups. The median PFS for the CA-TTF group was 11.9 months (95% CI: 8.9–20.4), and it was 7.5 months for the A-TTF group (95% CI: 6.0–NA, *P* = 0.85) (Fig. 2E). The median OS for the CA-TTF group was 18.1 months (95% CI: 14.6–NA), whereas it was 17.0 months for the A-TTF group (95% CI: 14.6-NA, *P* = 0.48) (Fig. 2F).

For subgroup analysis, age, sex, extent of resection, *MGMT* promoter methylation status, *TERT* promoter mutation status, baseline KPS score, and duration of TTFields therapy were evaluated. There was almost no difference in PFS or OS between CA-TTF and A-TTF groups (Supplementary Tables S1, S2). The median PFS values were 8.5 months (95% CI: 5.3–NA) for the A-TTF plus STR/Biopsy group, 13.0 months (95% CI: 4.9–NA) for the CA-TTF plus STR/biopsy group, 16.0 months (95% CI: 9.0–NA) for the A-TTF plus GTR group, and 17.1 months (95% CI: 11.1–NA) for the CA-TTF plus GTR group (*P* = 0.052) (Supplementary Figure S1A). The corresponding median OS values were 13.6 months (95% CI: 10.0–NA), 17.9 months (95% CI: 14.9–NA), 26.3 months (95% CI: 17.0–NA), and NA (95% CI: 17.0–NA), respectively (*P* = 0.043) (Supplementary Figure S1B).

## Discussion

This is the first retrospective study to compare the clinical benefits of CRT with or without concurrent TTFields therapy in patients with ndGBM in China. Our experience confirmed the feasibility and safety of concurrent TTFields therapy and CRT in the Chinese patient population.

The present study demonstrated that both the CA-TTF and the A-TTF groups exhibited similar low-grade skin toxicities, confirming the safety of TTFields therapy in combination with CRT. No significant differences were found in PFS or OS between the CA-TTF and A-TTF groups. However, the data confirmed the benefits of TTFields therapy, with a PFS of approximately 14 months and an OS of approximately 20 months, which were similar to the EF-14 trial and favorable compared to historical benchmarks, validating the benefits in the Chinese patient population.

Preclinical studies have shown a synergistic effect between TTFields therapy and RT, supporting the concurrent use of TTFields therapy with CRT. Our previous dosimetry study indicated that the dose distribution in the clinical target volume (CTV) varied by less than 1%, which is not clinically significant [22]. However, an elevated scalp dose was noted, aligning with other research [23]. Therefore, when applying TTFields therapy during RT, it is crucial to contour the scalp and define it as an OAR in radiation planning using constraints recommended by the SPARE trial. The elevated scalp dose remains a concern despite dosimetry studies indicating minimal impact on the CTV, requiring careful planning and monitoring.

Several clinical trials have investigated the safety and efficacy of concurrent TTFields therapy, which appeared feasible and well-tolerated [15, 17]. In the current study of 72 patients, 41 patients received concurrent TTFields therapy with CRT (CA-TTF group), and 31 patients received adjuvant TTFields therapy with TMZ following CRT (A-TTF group). Common but mild scalp irritation was found in about 55–60% of patients. Dermatitis, which included symptoms, such as scalp irritation, dry skin, folliculitis, erythema, color change, and rash, was the most common cutaneous AE. Pruritus was the second most frequently reported skin-related AE, while electric sensation and skin burning sensation rarely occurred. There was no significant difference in skin toxicity between the CA-TTF and A-TTF groups. One (2.4%) patient in the CA-TTF group experienced grade 3 skin toxicity, as this patient did not apply the scalp-preserving procedure and the transducer arrays were not removed during RT. The radiation plan was suspended, while the skin reaction was improved in three days using topical corticosteroid ointment. Other patients who suffered from scalp toxicity did not interrupt their radiation plan, while suspended TTFields therapy for about three days, and their skin reaction was also recovered using topical corticosteroids ointment and then restarted the TTFields therapy. The results of the present study confirmed that concurrent use of TTFields therapy and CRT was demonstrated to be safe and well-tolerated for patients with ndGBM.

Preclinical studies have indicated that TTFields therapy could inhibit DNA damage repair and promote cell death, potentially increasing the efficacy of RT [11]. The combination of TTFields therapy with CRT was associated with median PFS of 14.2 months in the CA-TTF group and 15.0 months in the A-TTF group. The OS was 20.8 months in the CA-TTF group versus 20.0 months in the A-TTF group (Fig. 1). After adjusting for baseline characteristics, the HRs for PFS and OS suggested a potential benefit for concurrent TTFields therapy, although the differences were not statistically significant.

Regarding molecular markers, the distribution of the MGMT promoter status was even between the two groups. MGMT promoter methylation is typically associated with a better prognosis [24]. In this study, a benefit in terms of PFS and OS was found in the *MGMT* promoter methylation group compared with the unmethylated group across all populations. Regarding *TERT* mutation, in the CA-TTF group, the proportion of *TERT* promoter mutation was 63.4%, which was higher than that in the A-TTF group (41.9%). Previous studies have reported an association between *TERT* promoter mutation and poor prognosis in patients with GBM [25]. The proportion of *TERT* promoter mutation was higher in the CA-TTF group, which was associated with a poorer prognosis, while these patients achieved survival rates comparable to the A-TTF group. These findings are exploratory and do not establish a causal relationship between *TERT* mutation status and the efficacy of concurrent TTFields therapy. Further research is required to validate this observation and elucidate underlying mechanisms.

The residual lesions in patients may affect the degree of benefit from the concurrent use of TTFields therapy. As illustrated in Figure S1A, the use of CA-TTF, compared with the A-TTF group, could improve PFS in the STR/biopsy subgroup (median PFS, 13 months vs. 8.5 months). In the GTR subgroup, the median PFS with the use of CA-TTF and A-TTF emerged similar. A similar trend was found in median OS. The *P*-values between the two groups in the figure are inappropriate for making comparison due to the limited number of samples. However, trends observed in both PFS and OS suggested that CA-TTF could promote control disease recurrence and positively impact patient survival outcomes in the presence of residual lesions. Further validation with a larger sample size is therefore required.

There were no significant differences in PFS or OS between the CA-TTF and A-TTF groups. Despite no significant differences in baseline characteristics between the two groups, the CA-TTF group had a higher proportion of *TERT* promoter mutations, indicating a potentially worse prognosis. The limited sample size could affect the statistical power to detect significant differences, necessitating larger prospective trials to validate these findings. Additionally, patients with early progression or clinical decline were excluded from the A-TTF group, which might influence survival outcomes. Also, compliance and corticosteroid use have been shown to significantly impact TTFields efficacy. Recent studies highlight the negative influence of high-dose dexamethasone and poor compliance on TTFields treatment outcomes in glioblastoma patients [26]. These findings underscore the importance of optimizing patient management to improve treatment efficacy.

## Limitations

The present study has several limitations, primarily due to its retrospective design, introducing potential patient selection bias. This method was selected in response to the inconsistent and limited insurance coverage for TTFields therapy. Additionally, the relatively small sample size of 72 patients and median follow-up time of 18.0 months (IQR: 12.1) might affect the robustness of the results, particularly as the survival data for patients still alive at the last follow-up are yet to be fully updated.

## Conclusions

This study provided the first real-world analysis examining the survival benefits of concurrent versus adjuvant TTFields therapy combined with CRT in patients with ndGBM. The findings suggested that TTFields, when used concurrently with CRT, could maintain a favorable safety profile, with no significant differences observed in survival outcomes compared with adjuvant therapy (A-TTF group). The study discussed clinical experiences with synchronous electric field therapy and highlighted that combining TTFields therapy with CRT could remain an investigational strategy for treating ndGBM. Ongoing follow-up data from the phase 3 trial (EF-32 NCT04471844) may further elucidate its potential advantages.

## Supplementary Information

Supplementary Material 1: Figure S1. Comparison of progression-free survival (A) and overall survival (B) between TTF plus extent of resection status. CA-TTF: concurrent and adjuvant TTFields therapy group; A-TTF: adjuvant TTFields therapy with temozolomide; GTR: gross tumor resection; STR: subtotal resection. Table S1. Progression-free survival for each prognostic patient subgroup treated with CA-TTF versus A-TTF group. Table S2. Overall survival among each prognostic patient subgroup treated with CA-TTF compared with the A-TTF group.

## Data Availability

The datasets used and/or analyzed during the current study are available from the corresponding author upon reasonable request.
