# Supplementary figures and images for: Chemoradiation treatment with or without concurrent tumor-treating fields (TTFields) therapy in newly diagnosed glioblastoma (GBM) patients in China
Source: Chin Neurosurg J. 2025 Mar 7;11:5. doi: 10.1186/s41016-025-00391-w (PMC11887109; doi:10.1186/s41016-025-00391-w)

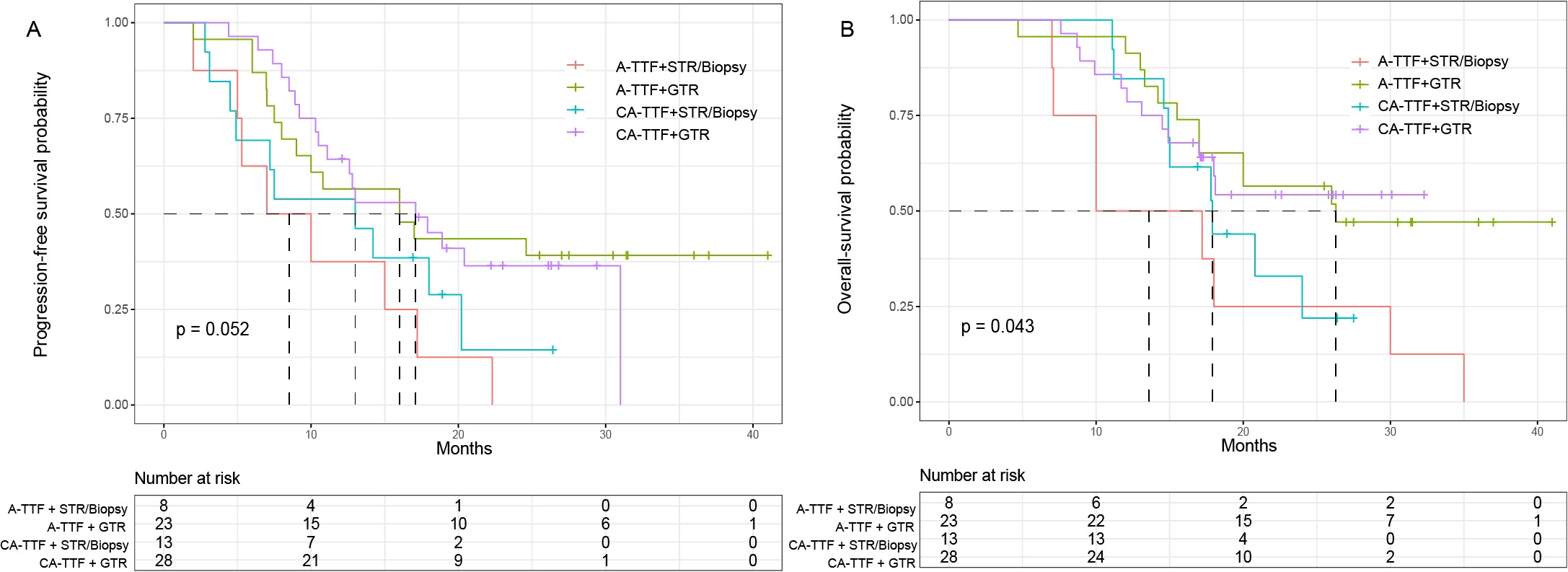

Supplement: Supplementary file 1 — Supplementary Material 1: Figure S1. Comparison of progression-free survival (A) and overall survival (B) between TTF plus extent of resection status. CA-TTF: concurrent and adjuvant TTFields therapy group; A-TTF: adjuvant TTFields therapy with temozolomide; GTR: gross tumor resection; STR: subtotal resection. Table S1. Progression-free survival for each prognostic patient subgroup treated with CA-TTF versus A-TTF group. Table S2. Overall survival among each prognostic patient subgroup treated with CA-TTF compared with the A-TTF group. [file 41016_2025_391_MOESM1_ESM.jpg]
